# Supplementary material for: Content-rich biological network constructed by mining PubMed abstracts
Source: BMC Bioinformatics. 2004 Oct 8;5:147. doi: 10.1186/1471-2105-5-147 (PMC528731; doi:10.1186/1471-2105-5-147)
Supplement: Additional File 5 — The original Chilibot query results of the term "long-term potentiation (LTP)" and 22 other terms, limiting the latest references analyzed to the years 1990, 1995, 2000, and 2004. [file 1471-2105-5-147-S5.bz2 › chilibotAdditionalFile5/ltp2000/html/PKC_KV4.2.html]

 


 **PKC** and **KV4.2** 
  
Found 3 abstracts in PubMed,  **3 abstracts were retrieved and analyzed**.  


---

 Search Google  |
 PDF files only 
|  EDU domain only 

---

**Interactive relationship** (e.g. stimulation, inhibition, etc)

- The voltage dependence and inactivation kinetics of  **Kv4.2**  were unchanged by  **PKC** , but small effects on the rates of inactivation and recovery from inactivation of native I to were observed.  Ref: 9362243 Am J Physiol, 1997

- :-)

  **Parallel relationship** (e.g. studied together, co-existance, homology, etc.)

  - Phorbol 12 myristate 13 acetate PMA suppressed both  **Kv4.2**  and Kv4.3 currents as well as native I to... chelerythrine.  Ref: 9362243 Am J Physiol, 1997
  - Phosphorylation consensus sites for various kinases exist within the sequence of the potassium channel subunit  **Kv4.2** , including consensus sites for extracellular signal regulated kinase mitogen activated protein kinase ERK MAPK, protein kinase A PKA, protein kinase C  **PKC** , and calcium calmodulin dependent kinase II CaMKII, and kinase assays have shown that particular amino acids of the consensus sites are bonafide phosphorylation sites in vitro.  Ref: 11040264 Learn MemLearn Mem, 1997
  - To determine whether protein kinase C  **PKC**  affects I to by an action on these molecular components, we compared the effects of  **PKC**  activation on  **Kv4.2**  and Kv4.3 currents expressed in Xenopus oocytes and rat ventricular I to.  Ref: 9362243 Am J Physiol, 1997
  - Thus  **Kv4.2**  and Kv4.3 proteins are important subunits of native rat ventricular I to, and  **PKC**  appears to reduce this current by affecting the molecular components of the channels mediating I to.  Ref: 9362243 Am J Physiol, 1997
